# Development of an Immune-Related Gene Signature for Prognosis in Melanoma

**DOI:** 10.3389/fonc.2020.602555

**Published:** 2021-01-21

**Authors:** Jia-An Zhang, Xu-Yue Zhou, Dan Huang, Chao Luan, Heng Gu, Mei Ju, Kun Chen

**Affiliations:** Institute of Dermatology, Jiangsu Key Laboratory of Molecular Biology for Skin Diseases and STIs, Chinese Academy of Medical Science and Peking Union Medical College, Nanjing, China

**Keywords:** melanoma, immune gene, tumor environment, prognostic, ssGSEA

## Abstract

Melanoma remains a potentially deadly malignant tumor. The incidence of melanoma continues to rise. Immunotherapy has become a new treatment method and is widely used in a variety of tumors. Original melanoma data were downloaded from TCGA. ssGSEA was performed to classify them. GSVA software and the "hclust" package were used to analyze the data. The ESTIMATE algorithm screened DEGs. The edgeR package and Venn diagram identified valid immune-related genes. Univariate, LASSO and multivariate analyses were used to explore the hub genes. The "rms" package established the nomogram and calibrated the curve. Immune infiltration data were obtained from the TIMER database. Compared with that of samples in the high immune cell infiltration cluster, we found that the tumor purity of samples in the low immune cell infiltration cluster was higher. The immune score, ESTIMATE score and stromal score in the low immune cell infiltration cluster were lower. In the high immune cell infiltration cluster, the immune components were more abundant, while the tumor purity was lower. The expression levels of TIGIT, PDCD1, LAG3, HAVCR2, CTLA4 and the HLA family were also higher in the high immune cell infiltration cluster. Survival analysis showed that patients in the high immune cell infiltration cluster had shorter OS than patients in the low immune cell infiltration cluster. IGHV1-18, CXCL11, LTF, and HLA-DQB1 were identified as immune cell infiltration-related DEGs. The prognosis of melanoma was significantly negatively correlated with the infiltration of CD4+ T cells, CD8+ T cells, dendritic cells, neutrophils and macrophages. In this study, we identified immune-related melanoma core genes and relevant immune cell subtypes, which may be used in targeted therapy and immunotherapy of melanoma.

## Introduction

Melanoma still remains a potentially deadly malignant tumor at the beginning of the 21st century. The incidence of melanoma unfortunately continues to rise, while the incidence of many tumor types is declining (1). Melanoma is mainly seen in young and middle-aged people, and the median age at diagnosis is 57 years old. It has been observed that the incidence increases linearly from 25 to 50 years old and then slows down, especially in women (2). Although most patients have localized disease at the time of diagnosis and are treated by surgically removing the primary tumor, many patients develop metastasis (3). It is generally understood that the normal function of a healthy immune system can protect and prevent the development of malignant tumors, and people with a genetically compromised immune system may have increased susceptibility to tumors (4). Immunotherapy has become a new treatment method and is widely used in a variety of tumors, such as gastric and esophageal cancer, pancreatic cancer and ovarian cancer (5–7). Experiments have shown that immune stimulation can participate in the treatment of melanoma (8). Targeted therapy for specific genes is also a research hotspot (9). Combining targeted therapy and immunotherapy is an important strategy to treat melanoma (10–12). Therefore, screening immune-related biological targets has become particularly important.

## Materials and Method

### Data Collection

RNA sequence and clinical data of melanoma were collected from TCGA (13). We downloaded the expression profiles of mRNAs (level 3) in cases including tumor tissues and normal tissues from TCGA database (http://cancergenome.nih.gov/) on april 15, 2019. The sequenced data were obtained from Illumina HiSeqRNASeq. The corresponding clinical information of patients was also downloaded from TCGA database. ssGSEA groups TCGA melanoma transcriptome data. From the results of Bindea et al (14), we used a set of marker genes for immune cell types. We utilized 29 immune data sets (including immune-related pathways, immune cell types and immune-related functions) and the ssGSEA method with the R software gene set variation analysis (GSVA) package to operate the related expression pathways, penetration levels of different immune cells and Activity of immune-related functions. The melanoma samples from TCGA were divided into low- and high- immune cell infiltration cluster by "hclust" package (15). GSE15605 from the GEO database including 58 melanoma samples was recruited for external validation.

### Verification of Effective Immune Grouping

The ESTIMATE algorithm was for identification of the differentially expressed genes (DEGs) in the melanoma expression profile data. The ESTIMATE algorithm was used to analyze the Immune Score, Stromal Score, Tumor Purity and ESTIMATE Score, and cluster heat maps and statistics were drawn for effective grouping.

### Selection of Immune-Related Genes in Melanoma

TCGA data was divided into high- and low- immune cell infiltration cluster. According to the standards of p <0.05 and| log2FC |> 2, we used the edgeR package to analyze DEGs. We used the same criteria to perform differential analysis on cancer groups and para-cancer groups to screen immune-related cancerous genes. The Venn diagram identified real immune-related genes from the above two analyses.

### Screen Prognostic Genes and Tap Their Characteristics

We utilized Univariate, lasso and multivariate analysis to dig out the correlation between the OS of patients and the expression level of immune-related genes. We calculated the regression coefficient and hazard ratio (HRs) of each gene, and finally the satisfactory mRNAs was identifed.

### Construct a Prognostic Model of Immune-Related Genes

The prognostic risk scoring model of melanoma patients in training cohort is a collection of each optimal prognosis mRNA expression level and relative regression coefficient weights calculated from the multivariate model as the following method:

$$Risk\;Score(patient)=\sum_{i}\text{Coefficient}({\text{mRNA}}_{i})\times \text{Expression}({\text{mRNA}}_{i})$$

Relying on the median risk score, all patients in the cohort were classified into high- and low-risk groups. Kaplan–Meier survival curves of the two groups were completed. We proposed ROC curves (16) to evaluate the specificity and sensitivity of the model. We also conducted a multivariate analysis of several clinical characteristics of melanoma patients to check the independence of the prognostic models without their clinical characteristics.

### Verify the Effect of Prognostic Models

With the cut-off values calculated from the training cohort, we compared the risk scores from the testing and entire cohort and then patient can be classified into high- or low-risk groups. Kaplan-Meier curve, Time-dependent ROC and Cox multivariate analysis were all conducted. Based on the clinicopathological characteristics, we conducted a stratification analysis of the entire cohort samples.

### Confirmation of Hub Immune Related Genes

The "rms" package established the nomogram and calibrate curve, checking the accuracy and the consistency index between the predicted probability and the actual observation frequency. We next displayed the results in the calibration curve, in order to represent the performance of nomogram.

### Analysis of Correlation With Immune Cell Infiltration

Immune infiltration data can be obtained from the tumor obtained from immune estimation resource (TIMER) database (17). We rely on the Pearson correlation coefficient to calculate the degree of correlation between immune infiltration and risk score. Meanwhile, we used the tumor-immune system interactions and drugbank (TISIDB) database to investigate the expression of these core immune-related genes in different molecular subtypes of cutaneous melanoma (18).

### RNA Extraction, cDNA Synthesis, and qRT-PCR

Total RNA was extracted respectively from melanoma cell line A375, A815, SK-MEL-28 and normal human epidermal melanocytes (NHEM) using TRIzol® reagent (Invitrogen, Carlsbad, CA) according to the manufacturer’s protocol. cDNA was synthesized using reverse transcription kit (TaKaRa Biotechnology, Shiga, Japan). RNA expression levels were detected using the SYBR Green Mix (TaKaRa Biotechnology, Shiga, Japan). Target gene expression values were normalized to human GAPDH. The primer sequences were as follows: GAPDH (forward: 5′‐ACTTTGGTATCGTGGAAGGACTA‐3′, reverse: 5′‐GTCTCTCTCTTCCTCTTGTGCTC‐3′); IGHV1-18((forward: 5′‐AACCAGGCCAGTCATGTGAG‐3′, reverse: 5′‐TGTAAGCGCTGATCCATCCC‐3′); CXCL11(forward: 5′‐GACGCTGTCTTTGCATAGGC‐3′, reverse: 5′‐GGATTTAGGCATCGTTGTCCTTT‐3′); LTF(forward: 5′‐AGTCTACGGGACCGAAAGACA‐3′, reverse: 5′‐CAGACCTTGCAGTTCGTTCAG‐3′); and HLA-DQB1(forward: 5′‐GCGGGATCTTGCAGAGGAG‐3′, reverse: 5′‐ACTTTGATCTGGCCTGGATAGAA‐3′).

## Results

### Differentiated Grouping of Melanoma Tissue

**Figure 1:**
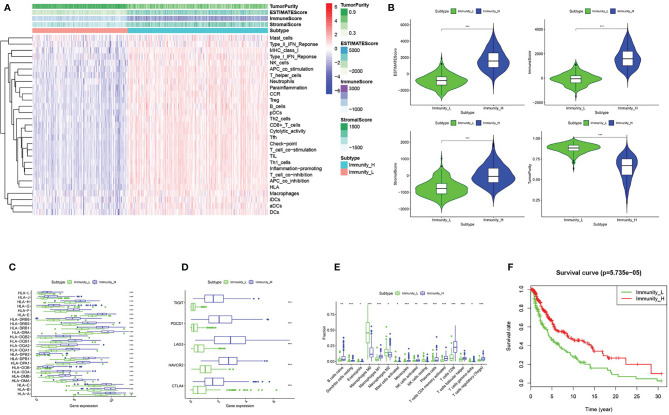
Grouping and verification of melanoma. **(A)** The immune cells were highly expressed in the high immune cell infiltration group (Immunity_H), and the low expression in the low immune cell infiltration group (Immunity_L). The Tumor Purity, ESTIMATE Score, Immune Score and Stromal Score were illustrated along with the grouping information. **(B)** There is a statistical difference of the Tumor Purity, ESTIMATE Score, Immune Score and Stromal Score between the high immune cell infiltration cluster and the low immune cell infiltration cluster **(C, D)** The expression of HLA family genes, TIGIT, PDCD1, LAG3, HAVCR2, and CTLA4 in the high immune cell infiltration cluster (red) were significantly higher than that of the low immune cell infiltration cluster (green) **(E)** The statistical graph shows the difference in the proportion of each immune cell between the high immune cell infiltration cluster (red) and the low immune cell infiltration cluster (green). **(F)** Survival difference between high immune cell infiltration cluster and low immune cell infiltration cluster. *P < 0.05, **P < 0.01, ***P < 0.001.

We obtained melanoma samples and normal skin tissue samples from the TCGA database. We used ssGSEA to analyze the transcriptome data of melanoma tissue samples to assess the immune cell infiltration state. After controlling for the enrichment of multiple immune cell types, melanoma samples were divided into high and low immune cell infiltration clusters according to the degree of immune infiltration (**Figure 1A**). To test the authenticity of the above grouping scheme, we used the ESTIMATE algorithm to analyze the expression profile of melanoma and calculated the immune score, ESTIMATE score, stromal score, and tumor purity. The results suggested that the tumor purity of the high immune cell infiltration group was lower than that of the low immune cell infiltration cluster. In contrast, the values of the ESTIMATE score, immune score and stromal score were higher in the high immune cell infiltration cluster than in the low immune cell infiltration cluster (**Figure 1A**). The box chart shows that the high immune cell infiltration cluster had significantly higher immune score, ESTIMATE score and stromal score and lower tumor purity than the low immune cell infiltration cluster (**Figure 1B**). There were more immune components in the high immune cell infiltration cluster than in the low immune cell infiltration cluster, but the tumor purity of the high immune cell infiltration cluster was lower, and the expression levels of TIGIT, PDCD1, LAG3, HAVCR2, CTLA4 and the HLA family were also higher in the high immune cell infiltration cluster (**Figures 1C, D**). The CIBERSORT method was used to analyze the above two clusters and showed that there were more types of immune cells in the high immune cell infiltration cluster (**Figure 1E**). Survival analysis demonstrated that patients from the low immune cell infiltration cluster had worse prognosis than patients in the high immune cell infiltration cluster (**Figure 1F**).

### Analysis of DEGs With High and Low Immune Cell Infiltration

**Figure 2:**
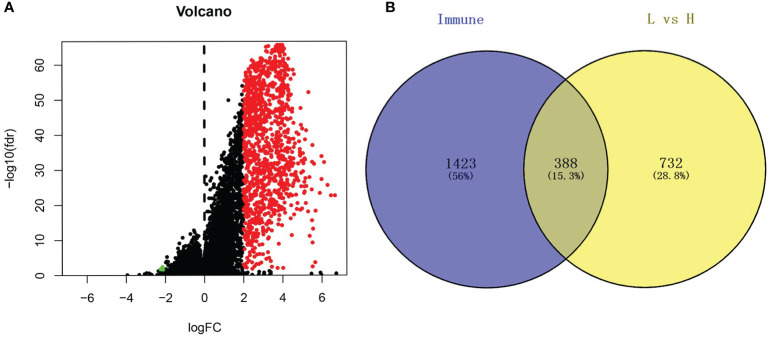
Analysis of differentially expressed genes. **(A)** The volcano graph shows the distribution of differential genes between high immune cell infiltration cluster and low immune cell infiltration cluster, red dots represent up-regulated genes, green dots represent down-regulated genes. **(B)** Using the Venn diagram to extract intersection points, we obtained a total of 388 differentially expressed genes.

Based on the cutoff, which was |log2FC| > 2 and FDR < 0.05, we identified 1120 DEGs between the low and high immune cell infiltration clusters, which included 1116 upregulated DEGs and 4 downregulated DEGs (**Figure 2A**). We conducted a Venn analysis based on the immune genes from the import database and the DEGs from the high and low immune cell infiltration clusters. Then, we found 388 overlapping genes (**Figure 2B**), which were considered to be real DEGs.

### Prognosis Models of Immune Cell Infiltration-Related DEGs

**Figure 3:**
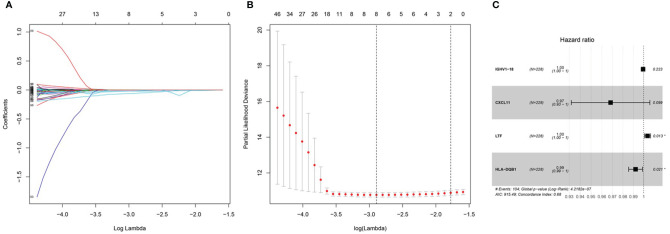
Prognosis model of training cohort. **(A, B)** LASSO Cox regression analysis of training cohort. **(C)** multivariate Cox proportional hazards regression analysis of training cohort.

**Figure 4:**
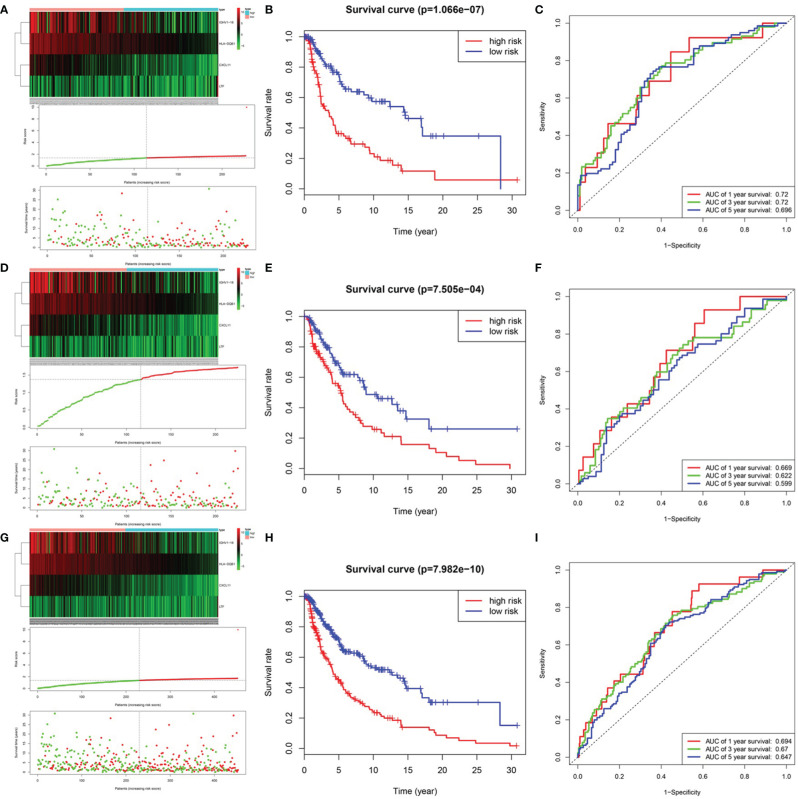
Prognosis model of training, testing and entire cohort. **(A)** The risk score and survival status of training cohort. **(B)** Survival analysis between low-risk patients and high-risk patients of training cohort. **(C)** ROC curve analysis of training cohort. **(D)** The risk score and survival status of testing cohort. **(E)** Survival analysis between low-risk patients and high-risk patients of testing cohort **(F)** ROC curve analysis of testing cohort. **(G)** The risk score and survival status of entire cohort. **(B)** Survival analysis between low-risk patients and high-risk patients of entire cohort **(H)** Survival analysis between low-risk patients and high-risk patients of training cohort **(I)** ROC curve analysis of entire cohort.

**Figure 5:**
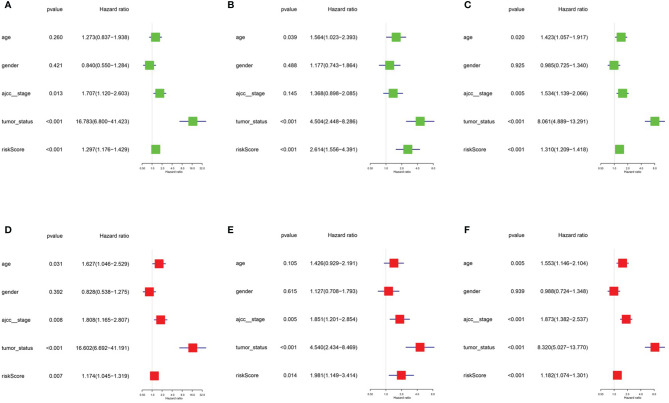
Univariate model and multivariate model of the training, testing and entire cohort. **(A)** Univariate model of training cohort. **(B)** Univariate model of testing cohort **(C)** Univariate model of entire cohort **(D)** multivariate model of training cohort **(E)** multivariate model of testing cohort **(F)** multivariate model of entire cohort.

**Figure 6:**
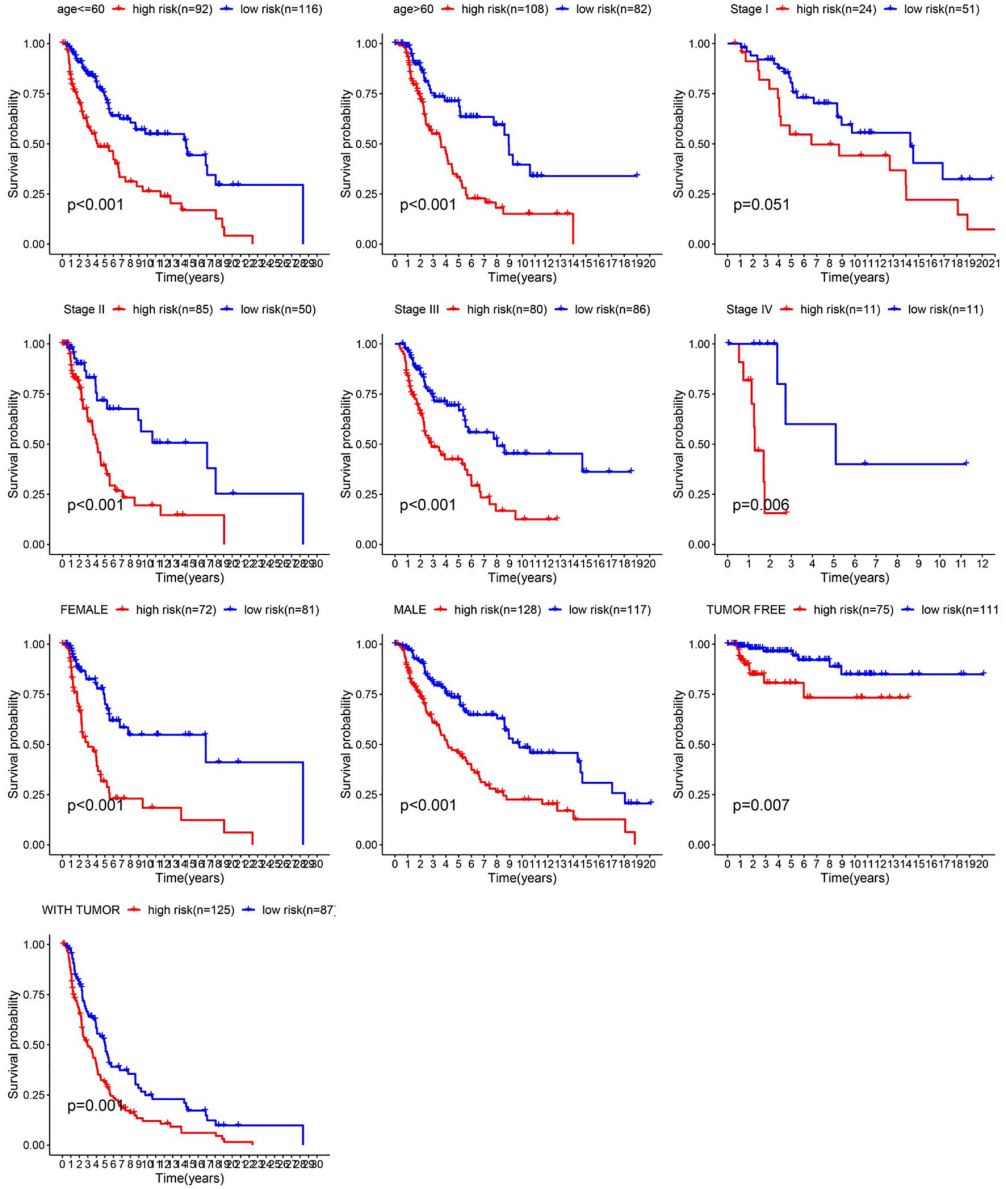
Survival time of patients in high-risk and low-risk cluster of different subgroups.

After integrating clinical information into gene expression profiles, we obtained 453 samples. We randomly selected 228 samples as the training cohort and the remaining 225 samples comprised the test cohort. All the samples together are referred to as the entire cohort. Then, we built a prognostic model with each cohort. In the training cohort, based on p < 0.05, univariate Cox regression analysis identified 171 genes (**Table 1**). The LASSO Cox regression algorithm was performed next (**Figures 3A, B**). Finally, multivariate Cox proportional hazards regression analysis was conducted, and the risk scores were calculated (**Figure 3C**). IGHV1-18, CXCL11, LTF and HLA-DQB1 were identified as immune cell infiltration-related DEGs. The risk score was calculated using the following formula: -0.000600085×IGHV1-18-0.032242183×CXCL11+0.003776394×LTF-0.007893899×HLA-DQB1. The survival status and risk score calculated by the prognostic model are illustrated in **Figure 4A**. Samples were classified into low- and high-risk clusters according to the median risk score. Survival analysis indicated that low-risk patients had significantly longer overall survival times than high-risk patients (**Figure 4B**). ROC curve analysis showed that the specificity and sensitivity were highest when the risk score was 0.72, 0.72, and 0.696 according to the 1-, 3-, and 5-year survival of the area under the receiver operating characteristic curve (AUC) value, respectively (**Figure 4C**). For the testing cohort, the risk score and survival status indicated by the prognostic model are displayed in **Figure 4D**. Samples were divided into low- and high-risk clusters according to the median risk score. Survival analysis indicated that low-risk patients had significantly longer overall survival times than high-risk patients (**Figure 4E**). ROC curve analysis showed that the specificity and sensitivity were highest when the risk score was 0.669, 0.622, and 0.599 according to the 1-, 3-, and 5-year survival of the area under the AUC value, respectively (**Figure 4F**). For the entire cohort, the risk score and survival status are illustrated in **Figure 4G**. Samples were classified into low- and high-risk clusters according to the median risk score. Survival analysis indicated that low-risk patients had significantly longer overall survival times than high-risk patients (**Figure 4H**). ROC curve analysis showed that the specificity and sensitivity were highest when the risk score was 0.694, 0.67, and 0.647 according to the 1-, 3-, and 5-year survival of the area under the AUC value, respectively (**Figure 4I**). The univariate model of the training, testing and entire cohorts is shown in **Figures 5A–C**, while the multivariate model of the training, testing and entire cohorts is shown in **Figures 5D–F**. The results all demonstrated that the prognostic model has independent and moderate prognostic power for immune cell infiltration. Taking the median risk score as the standard, we divided the sample of the entire cohort into a high-risk cluster and a low-risk cluster. Based on different clinical factors, we conducted a survival analysis of the two groups of samples. In the subgroup analysis stage II, stage III, stage IV, age ≤ 60, age > 60, female, male, with tumor and free of tumor, patients in the high-risk group had shorter overall survival times than those in the low-risk group (**Figure 6**).

**Table 1:** Univariate Cox proportional hazards regression analysis.

id	HR	HR.95L	HR.95H	pvalue
CXCL13	0.984348	0.973053	0.995773	0.007377
IGLC3	0.999731	0.999385	1.000076	0.126396
LTA	0.718796	0.561118	0.920782	0.008971
IL21R	0.800788	0.691007	0.92801	0.003146
LYZ	0.999125	0.997604	1.000648	0.259777
TRAV17	0.826178	0.681196	1.002016	0.052439
CD79A	0.99371	0.985742	1.001743	0.124528
CD8A	0.983734	0.969986	0.997677	0.02238
TRAV24	0.821879	0.652012	1.036	0.0968
IGHD3-9	0.972616	0.929259	1.017996	0.232728
IGLV5-48	0.91922	0.788898	1.071071	0.280237
TRAV1-1	0.649291	0.420767	1.00193	0.051027
IGKV5-2	0.997661	0.98566	1.009809	0.704548
IRF1	0.963002	0.942933	0.983497	0.00045
TRAV9-2	0.924825	0.794167	1.076979	0.314579
TNFSF10	0.962967	0.935795	0.990928	0.009766
IGKV1D-42	1.035907	0.9516	1.127684	0.415348
IGLV7-43	0.995479	0.988942	1.002059	0.177635
CD72	0.900216	0.837435	0.967703	0.004371
IGKV1D-13	0.998284	0.992279	1.004325	0.576791
IGKV3D-20	0.985753	0.97008	1.001679	0.079284
IGLV3-22	0.289776	0.094598	0.887653	0.030112
TRBJ2-2	0.830183	0.688906	1.000433	0.050534
IGKV1-6	1.000115	0.999664	1.000567	0.616898
TRBV20-1	0.971925	0.941046	1.003817	0.083868
CHIT1	1.005764	0.980735	1.031431	0.654887
CCL19	0.997498	0.99449	1.000516	0.104117
TRBV5-6	0.812817	0.679419	0.972406	0.023458
TRAV20	0.661623	0.456921	0.958033	0.028746
HCST	0.970771	0.952567	0.989323	0.002131
IL21	0.170536	0.026202	1.10993	0.064194
TRAV12-3	0.888752	0.777468	1.015966	0.084007
IGHV3-23	0.99981	0.999441	1.000179	0.313097
CXCR5	0.467469	0.162574	1.344173	0.158217
GNLY	0.969729	0.931052	1.010013	0.138824
TRAV4	0.850566	0.739477	0.978344	0.023417
SH2D1A	0.919189	0.857848	0.984917	0.01679
TRBJ2-7	0.936429	0.882962	0.993132	0.028545
TRAV12-2	0.934148	0.847216	1.03	0.17167
TRBC2	0.987932	0.979453	0.996485	0.00577
IGHA2	1.000057	0.997625	1.002495	0.963297
TRAV2	0.84817	0.710529	1.012474	0.068342
IGHV1-18	0.998585	0.997382	0.999791	0.021452
CTSS	0.988036	0.980716	0.995411	0.001512
PRF1	0.988879	0.977689	1.000196	0.054079
CXCL11	0.932669	0.898252	0.968404	0.00028
SECTM1	0.958286	0.928803	0.988705	0.007531
PTPN6	0.963732	0.939021	0.989093	0.005312
TRDV3	0.241489	0.022423	2.600813	0.241296
IDO1	0.983781	0.969629	0.998139	0.026967
PTPRC	0.976049	0.955428	0.997116	0.026074
IGLV4-69	0.99909	0.997747	1.000434	0.184465
TRAV26-2	0.700281	0.510627	0.960374	0.027045
IGKV3D-11	0.944423	0.88656	1.006062	0.076294
TRAV14DV4	0.89659	0.747191	1.07586	0.240504
IGLV3-16	0.955149	0.892531	1.022159	0.184697
IGLV1-40	0.999581	0.999035	1.000127	0.132357
GZMB	0.976692	0.959624	0.994064	0.008746
IGKV3D-7	0.78275	0.484053	1.265766	0.317857
IGHD	0.991429	0.974885	1.008254	0.316063
IL34	0.999049	0.994983	1.003132	0.647628
IGHA1	0.999931	0.999704	1.000158	0.549881
TRAJ5	0.687367	0.427064	1.10633	0.122629
IGLV3-10	0.999536	0.998487	1.000586	0.385935
IGHV3-64	0.984209	0.957531	1.011629	0.256252
KIR2DL3	0.019139	0.000305	1.1992	0.060942
IGHV4-34	0.996772	0.99374	0.999813	0.037507
IGHV3-38	0.968389	0.866286	1.082527	0.572049
IGHV4-31	0.995077	0.988644	1.001551	0.135798
IGKV1D-12	0.992894	0.93578	1.053494	0.813484
IGKV1-12	0.994344	0.978067	1.010892	0.500597
IGHV3-7	0.982059	0.946401	1.01906	0.337349
CD48	0.961365	0.934992	0.988482	0.005499
IGHD2-2	0.992012	0.967391	1.017258	0.531639
KIR3DL1	0.002304	1.80E-05	0.295324	0.014187
BLNK	0.834012	0.717151	0.969915	0.018445
IGHV1-24	0.99813	0.995787	1.000479	0.118614
TRBV11-3	0.411952	0.183004	0.927328	0.032177
IGHV3-11	0.999077	0.996708	1.001452	0.445862
RARRES3	0.989528	0.983129	0.995968	0.00147
TRAV35	0.798858	0.563439	1.132643	0.207406
IGKV2D-28	0.974061	0.91315	1.039035	0.425044
XCL1	0.712237	0.555962	0.91244	0.007253
TRAV25	0.656972	0.425497	1.014373	0.058016
IGKV1-5	0.999478	0.998807	1.00015	0.127654
CD19	0.944771	0.876754	1.018063	0.136132
TRBV11-1	0.549395	0.274752	1.098574	0.090255
SOCS1	0.892873	0.830715	0.959683	0.002086
CYBB	0.980011	0.966519	0.993692	0.004308
IGHV7-81	1.005581	0.977871	1.034076	0.69627
TRBV19	0.899731	0.82719	0.978633	0.013757
IFNG	0.860939	0.76748	0.965778	0.010653
IGHV2-5	0.986947	0.971414	1.002729	0.104539
CCR3	2.76E-05	1.00E-08	0.075664	0.009351
CCL25	0.322467	0.109665	0.948202	0.039723
PTAFR	0.915076	0.861884	0.971552	0.003678
IGKV2-28	0.970064	0.91002	1.034069	0.351177
IL27	0.245756	0.10748	0.561927	0.000881
IGHV3-49	0.994136	0.987745	1.000569	0.073918
IGHD3-22	0.982886	0.93641	1.031669	0.48489
IGHV2-70	1.000499	0.999137	1.001863	0.472696
IGHG1	0.999821	0.999656	0.999985	0.032441
TRAV36DV7	0.587123	0.377844	0.912319	0.017883
IGKV1-13	1.001958	0.972058	1.032776	0.899314
IGKV1-27	0.996958	0.992173	1.001767	0.21461
IGKV3-7	1.002734	0.982339	1.023553	0.794533
IGHG2	1.000014	0.999913	1.000115	0.784598
TRAV3	0.827716	0.690175	0.992667	0.041416
TRAV26-1	0.851723	0.645984	1.122988	0.255242
RAC2	0.991108	0.983664	0.998608	0.020225
IGLV2-33	0.902229	0.647986	1.256227	0.542373
TRGV9	0.004802	4.81E-05	0.479311	0.023021
PNOC	0.81392	0.63008	1.0514	0.11497
NCR3	0.766893	0.609303	0.965241	0.023735
CCL4	0.96172	0.935473	0.988702	0.005696
TRGC2	0.864399	0.760495	0.9825	0.025737
CD28	0.956191	0.852977	1.071895	0.442093
TNFSF8	0.783604	0.616645	0.995767	0.04608
TRBC1	0.78364	0.629949	0.974826	0.028607
CR2	0.954569	0.896751	1.016114	0.144698
TRAV39	0.770688	0.539998	1.099931	0.15124
IGKV2-24	0.998599	0.996005	1.001199	0.290712
TRBV6-6	0.842065	0.70615	1.00414	0.055625
IGLV7-46	1.000504	0.989546	1.011583	0.928594
ITK	0.847464	0.736534	0.975101	0.020766
CXCR3	0.963645	0.931746	0.996635	0.031067
TRAV8-4	0.770147	0.606726	0.977585	0.031852
TRBV6-1	0.854175	0.742895	0.982124	0.026881
CD1B	0.558271	0.298965	1.042485	0.067342
TNFSF14	0.409643	0.212892	0.788226	0.007527
TRAJ3	0.871951	0.662436	1.147732	0.328445
IGHV3-35	0.93667	0.819885	1.07009	0.335587
HLA-DRB5	0.997537	0.995973	0.999103	0.002066
IL32	0.981307	0.966859	0.995971	0.012652
TNFRSF18	0.80822	0.705821	0.925475	0.002067
CXCL9	0.995984	0.993101	0.998876	0.006531
IGLV1-50	0.901099	0.774313	1.048646	0.178295
IGKV2D-30	0.975436	0.906451	1.04967	0.506311
TRAV22	0.638368	0.423484	0.96229	0.03207
IL7R	0.968323	0.928411	1.009952	0.133912
FCGR3A	0.98762	0.979817	0.995486	0.002085
IGKV1D-16	0.978818	0.94709	1.011609	0.202856
TRAV23DV6	0.653167	0.440552	0.968392	0.034021
CLEC4M	0.608771	0.252406	1.468279	0.269204
IGHV4-4	0.983161	0.960424	1.006436	0.154864
TRBV7-6	0.757145	0.604004	0.949112	0.015823
IGHJ3	0.997271	0.992332	1.002236	0.28083
TRBV10-3	0.840569	0.734592	0.961835	0.01154
IGHG4	0.99985	0.999522	1.000178	0.369527
IGHV6-1	0.99903	0.976513	1.022067	0.933511
TRAV1-2	0.891762	0.747441	1.06395	0.203453
TRAV8-3	0.886507	0.780229	1.007262	0.064471
IGKV1D-39	1.001275	0.995844	1.006735	0.646172
IGHV4-28	0.998921	0.99413	1.003735	0.659886
TRDV1	0.736006	0.544399	0.995052	0.046349
CCR5	0.937949	0.896262	0.981574	0.00575
HLA-DMA	0.989833	0.984344	0.995353	0.000316
IGLV3-27	0.994248	0.981759	1.006897	0.371138
IGHV1-45	1.002258	0.996857	1.007688	0.41334
HLA-DOA	0.972465	0.956169	0.989039	0.001203
IL2RA	0.88327	0.801561	0.973308	0.012202
CD1E	0.622906	0.404843	0.958428	0.031311
XCL2	0.843041	0.757858	0.937797	0.00168
HLA-DRA	0.999395	0.999083	0.999706	0.000141
IGLV8-61	0.99894	0.994593	1.003307	0.633755
VAV1	0.919914	0.865543	0.977701	0.007243
IGHV1-2	0.999971	0.999682	1.000259	0.842042
IGLV5-45	0.997252	0.990007	1.00455	0.459541
IGLV2-8	0.996932	0.99271	1.001171	0.155837
FLT3	0.436393	0.207338	0.918495	0.028971
PRKCQ	0.842273	0.716929	0.989531	0.0368
IGKV2D-24	1.004247	0.92161	1.094294	0.922937
IGHG3	0.999201	0.99834	1.000063	0.069351
IGHV4-59	0.99918	0.997748	1.000615	0.262682
IGLC6	0.79439	0.665276	0.948563	0.010975
IGKV1D-8	1.001325	0.996262	1.006415	0.608671
CCL5	0.996567	0.993823	0.99932	0.014543
IGLV6-57	0.997351	0.994576	1.000134	0.06212
IGHV1-58	0.998696	0.995351	1.002052	0.44587
ITGAL	0.970307	0.946315	0.994907	0.018291
IGKV6D-21	0.998215	0.991737	1.004736	0.590799
IGLC2	0.999607	0.999248	0.999967	0.032221
IGKJ5	0.993873	0.977175	1.010857	0.477168
ITGB2	0.987468	0.977836	0.997195	0.011681
CMKLR1	0.999697	0.980758	1.019001	0.975215
FGR	0.911703	0.851101	0.97662	0.008437
TRBJ2-3	0.891422	0.810397	0.980547	0.018078
IGLV2-18	1.001432	0.993677	1.009247	0.718248
TRBV4-2	0.93851	0.865915	1.017193	0.122354
TRAV29DV5	0.786441	0.644109	0.960225	0.018353
TRAV41	0.830592	0.646538	1.067042	0.146428
TRBV3-1	0.835045	0.720849	0.967332	0.016277
GPR33	0.007939	6.37E-06	9.8891	0.18357
IGLV3-1	0.99919	0.997691	1.000691	0.290134
TRBV7-3	0.719028	0.559452	0.92412	0.009986
CCR8	0.556926	0.297076	1.044065	0.067929
LTF	1.003849	1.000802	1.006904	0.013244
HLA-DQA2	0.993171	0.987225	0.999154	0.025332
TRBV7-7	0.853551	0.529137	1.376863	0.51629
INPP5D	0.931525	0.879298	0.986853	0.015974
CCL4L2	0.96138	0.918554	1.006202	0.090259
IGHV3-73	1.000297	0.9991	1.001496	0.626649
TRAC	0.990849	0.98393	0.997816	0.010127
CD1C	0.903242	0.800211	1.019537	0.09959
CYSLTR1	0.479634	0.261508	0.879701	0.01759
CCL8	0.913252	0.867414	0.961512	0.000553
IL2	0.046599	0.000773	2.809707	0.142642
ICOS	0.848494	0.733654	0.981311	0.026813
HLA-DOB	0.857442	0.776066	0.947351	0.002502
IGLV3-21	0.999785	0.999371	1.000199	0.309226
TNFRSF13C	0.951518	0.877325	1.031985	0.230204
FASLG	0.857629	0.762338	0.96483	0.010596
TRBV5-4	0.792528	0.644195	0.975017	0.02786
CD4	0.983991	0.972281	0.995842	0.008239
LTB	0.980002	0.962611	0.997708	0.027026
DES	1.000368	0.999574	1.001162	0.364319
CD3D	0.980379	0.966894	0.994051	0.005043
IGKV1-33	0.995864	0.954131	1.039422	0.849491
IGLV1-36	0.991292	0.979571	1.003153	0.149511
TRAV13-2	0.670833	0.493213	0.912419	0.010959
IGLV4-60	0.996695	0.990285	1.003146	0.314602
TRAV19	0.891057	0.808903	0.981555	0.019429
PTGDR	0.105933	0.021996	0.510174	0.005125
TRAV16	0.750522	0.573039	0.982975	0.037097
TRAV38-1	0.763484	0.528509	1.102929	0.150451
PDCD1	0.951432	0.914032	0.990362	0.014962
IGLV3-25	0.998951	0.997816	1.000087	0.070359
CD3E	0.983987	0.971234	0.996906	0.015286
IGHV5-51	0.998909	0.997801	1.000019	0.054012
IGLV1-44	0.999146	0.997812	1.000482	0.210314
KIR2DS4	0.395255	0.123802	1.261908	0.117067
TRAV10	0.731238	0.483965	1.10485	0.137157
CXCR6	0.93358	0.875641	0.995354	0.035516
PRKCB	0.968932	0.897984	1.045486	0.415955
TRAJ1	0.687138	0.478207	0.987354	0.042481
HLA-DQB1	0.987582	0.981567	0.993635	6.11E-05
IGLV1-47	1.000034	0.999873	1.000194	0.68076
IGKV1D-33	1.012493	0.97154	1.055172	0.555619
PTGER2	0.736064	0.549563	0.985856	0.039829
IGKV1-9	1.000002	0.999348	1.000657	0.995328
CCR7	0.972217	0.941698	1.003724	0.083358
IL2RG	0.985634	0.974714	0.996675	0.010901
TRGC1	0.444086	0.210248	0.937998	0.033359
CD3G	0.90894	0.834923	0.98952	0.02759
TRBV10-1	1.013973	0.762105	1.349081	0.92412
IGHV3-13	0.978668	0.953074	1.00495	0.11077
TRAV30	0.598581	0.358763	0.998706	0.049423
IGHV3-15	1.000011	0.999379	1.000643	0.972605
TRAV8-1	0.748404	0.534113	1.048671	0.092211
IGLV9-49	0.99951	0.997646	1.001378	0.607
HLA-DPA1	0.994536	0.991677	0.997403	0.000191
TRBJ2-1	0.9131	0.84178	0.990463	0.028459
IGLV3-12	0.928852	0.842442	1.024124	0.138479
CD247	0.898749	0.831399	0.971555	0.00723
IGLJ1	0.829772	0.628275	1.095891	0.18858
HLA-DPB1	0.996537	0.99467	0.998407	0.000288
IL12RB1	0.870102	0.799937	0.946422	0.00118
HLA-DRB1	0.998863	0.998319	0.999407	4.27E-05
IGHJ2	0.994165	0.983602	1.004841	0.28289
TLR8	0.853271	0.739648	0.984349	0.029531
TNFRSF13B	0.807552	0.572644	1.138823	0.222938
IGHE	0.938187	0.841135	1.046437	0.25211
TRAV8-6	0.782565	0.642955	0.95249	0.014467
IGHV3-21	0.999647	0.998987	1.000308	0.295134
TRBV10-2	1.032769	0.92244	1.156293	0.575909
IGHV4-61	0.973169	0.948904	0.998055	0.034766
IGKV1D-17	1.000348	0.998996	1.001703	0.61404
IGLV3-19	0.999794	0.999433	1.000155	0.263626
IL12B	0.00779	0.000143	0.423188	0.017226
HLA-DQA1	0.983727	0.975499	0.992025	0.000129
TRBV15	0.629736	0.436528	0.908458	0.013381
TRBV28	0.979161	0.96181	0.996825	0.020967
IGHV3-43	0.994268	0.98251	1.006166	0.343573
IGLV1-51	1.000029	0.999947	1.000111	0.486773
XCR1	0.763403	0.537731	1.083784	0.131056
IGKV1-39	0.98647	0.943986	1.030866	0.54418
TYROBP	0.995021	0.991542	0.998513	0.005232
TRBV7-4	0.745151	0.441872	1.256585	0.269886
LCK	0.962643	0.934168	0.991985	0.012946
TRBV9	0.899328	0.825984	0.979185	0.014502
IGHV2-26	0.99669	0.990276	1.003145	0.314161
CCR9	1.498761	0.376537	5.965639	0.565886
IGKV3-20	0.999422	0.998877	0.999967	0.037554
CD8B	0.963847	0.9336	0.995074	0.023604
TRBV30	0.822089	0.665262	1.015886	0.069674
SCGB3A1	1.009583	1.000513	1.018735	0.03833
CD40LG	0.844627	0.693676	1.028427	0.092776
IGHD3-3	1.000226	0.991038	1.0095	0.961674
MARCO	0.990272	0.977431	1.003283	0.142145
TNF	0.744613	0.575395	0.963596	0.024968
TRAV13-1	0.924656	0.835519	1.023303	0.129882
IGLV2-23	0.999573	0.998905	1.000241	0.209806
CD74	0.999549	0.999305	0.999792	0.000283
IGHV1-69	0.998389	0.995399	1.001388	0.292067
CSF2RB	0.914809	0.857012	0.976503	0.007495
IGHV3-20	0.994732	0.982661	1.006951	0.39648
IL18	0.908756	0.851412	0.969962	0.004014
CCRL2	0.749188	0.591398	0.949077	0.016707
TRBV2	0.862911	0.728245	1.022478	0.088533
IGLV10-54	0.966349	0.927966	1.006318	0.097848
TNFRSF1B	0.981432	0.967345	0.995723	0.011051
KIR2DL4	0.667522	0.51547	0.864427	0.00218
C3	0.993528	0.987138	0.99996	0.048595
KLRD1	0.476615	0.281335	0.807443	0.005866
IGLJ3	0.573863	0.331941	0.9921	0.046772
EBI3	0.925119	0.871703	0.981808	0.010317
TRBV18	0.791619	0.658424	0.951758	0.012919
IGHV3-53	0.999341	0.994257	1.004451	0.800085
IGKV2-30	0.992261	0.977885	1.006848	0.296785
IGLJ2	0.923443	0.83405	1.022417	0.125228
PIK3CG	0.97465	0.884587	1.073884	0.603738
IGHV1-46	0.998067	0.995325	1.000817	0.168072
IGHV3-74	0.999289	0.996461	1.002125	0.622735
IGHV1-3	0.99012	0.976618	1.003809	0.156401
TRBJ2-4	0.676673	0.471436	0.97126	0.034166
IGKV1D-43	1.004241	0.973598	1.035849	0.788953
TRBV29-1	0.946347	0.892433	1.003518	0.065384
IGKV3-11	0.999853	0.999454	1.000251	0.46783
IGKC	0.999876	0.999728	1.000023	0.098663
TRDC	0.769485	0.650771	0.909856	0.002177
IGKV1-16	0.999835	0.998703	1.000968	0.774937
TRBV12-4	1.007399	0.985876	1.029392	0.50349
IGKV4-1	0.999419	0.998872	0.999966	0.03751
ZAP70	0.922358	0.865082	0.983428	0.013479
IGKV2D-29	0.999748	0.996581	1.002925	0.876288
IGLV3-9	0.997901	0.993281	1.002543	0.374931
KIR3DL2	0.026264	0.001736	0.39737	0.008645
CCL22	0.91853	0.803383	1.050181	0.21368
CXCL10	0.993961	0.990185	0.997752	0.001816
IL10RA	0.956127	0.923944	0.98943	0.010222
TRBV6-5	0.932299	0.856419	1.014903	0.105567
HLA-DMB	0.964458	0.945515	0.98378	0.000349
TRAV6	0.652322	0.42155	1.009429	0.055132
TRBV12-5	0.62701	0.334467	1.175427	0.145431
IGKV3-15	0.99935	0.99849	1.000211	0.139011
TRBV27	0.669834	0.50164	0.894422	0.006602
PMCH	0.587635	0.151747	2.2756	0.441512
IGLV2-11	0.999238	0.998003	1.000475	0.22729
INSL3	0.264349	0.11061	0.631775	0.002762
IL2RB	0.973233	0.950341	0.996677	0.025479
IGLV2-14	0.999765	0.999344	1.000186	0.273154
IGHV4-39	0.999588	0.998904	1.000273	0.238744
CIITA	0.880804	0.819196	0.947045	0.000602
IGHV3-66	0.993763	0.979889	1.007833	0.383061
TRBV13	0.733836	0.58598	0.918999	0.007023
CELA1	0.017899	0.00026	1.233778	0.062505
IGHV3-48	0.997463	0.99359	1.00135	0.200473
TRBV4-1	0.945288	0.853279	1.047218	0.281522
CD79B	0.990187	0.971681	1.009046	0.305611
IL15RA	0.876656	0.792057	0.970291	0.011008
TRAV21	0.870157	0.783858	0.965956	0.009056
TRAV8-2	0.799841	0.65272	0.980122	0.031274
TRGV2	0.659298	0.429353	1.012392	0.056952
TRAV27	0.597365	0.382695	0.932453	0.023342
TRAV5	0.83798	0.67931	1.033712	0.098863
IGHJ1	0.981717	0.947547	1.01712	0.307336
CCR4	0.847081	0.667148	1.075544	0.173135
IL18RAP	0.449836	0.251667	0.80405	0.007018
TRBV7-9	0.9426	0.896452	0.991122	0.020991
TRBV12-3	0.738332	0.518661	1.051043	0.092246
TNFRSF17	0.928495	0.857256	1.005655	0.068529
IL9R	0.152866	0.018961	1.232431	0.07778
IGLC7	0.98671	0.966924	1.0069	0.19546
CD86	0.894026	0.840241	0.951254	0.000402
IGKV1-17	0.999297	0.997911	1.000685	0.320906
IL22RA2	0.035078	0.000723	1.702522	0.090774
TRAV12-1	0.900541	0.800563	1.013006	0.081028
CCL21	0.999681	0.999065	1.000298	0.311412
TRBV5-1	0.871976	0.775543	0.980399	0.021963
CARD11	0.90201	0.832149	0.977735	0.012162
TRBV14	0.694339	0.494855	0.974238	0.034772
KLRC1	0.390563	0.187834	0.8121	0.011828
IGLV5-52	0.811665	0.345463	1.907003	0.632089
HCK	0.955146	0.924763	0.986527	0.005397
IGHM	0.999505	0.998903	1.000108	0.107537
IGHV3-30	0.998836	0.997389	1.000285	0.115289
TLR7	0.806583	0.669315	0.972002	0.023926
IGKV2D-40	0.996216	0.988696	1.003792	0.326674
TRBV11-2	0.834541	0.694854	1.002309	0.052956
TRAV34	0.418904	0.159133	1.102731	0.078079
TRBV5-5	0.655048	0.453642	0.945874	0.02402
KIR2DL1	0.038509	0.001678	0.883633	0.041615
IGHV3-33	0.999745	0.998291	1.0012	0.730882
IGHV3-72	0.997166	0.992445	1.001909	0.241071
IGKV1-8	0.990277	0.973341	1.007508	0.266967
CCR6	0.151986	0.008686	2.65949	0.197003
IGKV6-21	0.999792	0.998969	1.000616	0.620933
TNFRSF9	0.848128	0.734197	0.979739	0.025216

### Construction of the Predictive Nomogram

**Figure 7:**
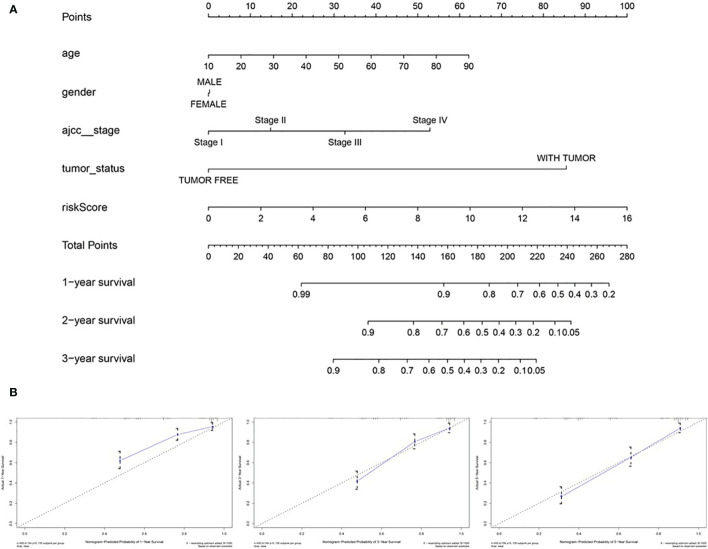
The nomogram of predicting 1-, 3‐, or 5‐year OS and prognostic value of 4 genes in the entire set. **(A)** The nomogram for predicting 1-, 3‐, or 5‐year OS. **(B)** The calibration plots for predicting 1-, 3‐ or 5‐ year OS. Nomogram‐predicted probability of survival is plotted on the x‐axis; actual survival is plotted on the y‐axis.

**Figure 8:**
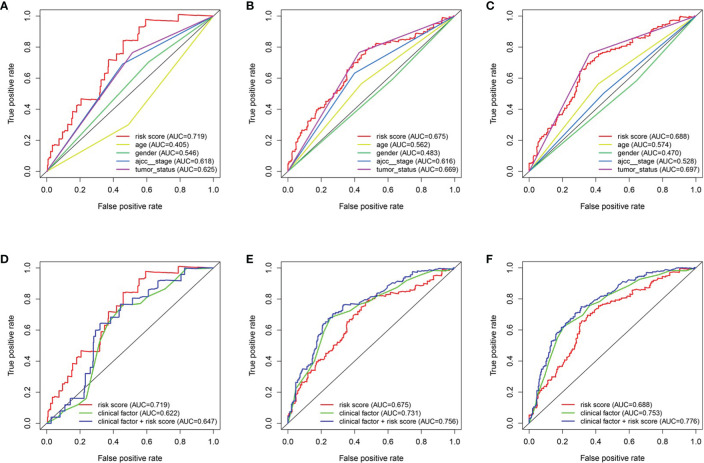
The relationship between four genes mRNA signature and different clinical features. **(A, D)** training cohort. **(B, E)** testing cohort. **(C, F)** entire cohort.

To predict the survival rate of melanoma patients from a clinical point of view, we constructed a nomogram using TCGA data to estimate the likelihood that the OS will last for 1, 3, and 5 years. We used the following six independent prognostic factors to predict the nomogram: age, AJCC stage, grade, histological type, risk score and tumor status (**Figure 7A**). The calibration chart shows that the effectiveness of the nomogram was very good, and the 45° line represents the best predicted case. (**Figure 7B**). ROC curve analysis illustrated that the 1-, 3-, and 5-year risk score AUC values were 0.719, 0.675 and 0.688, respectively. The AUC values for the 1-, 3- and 5-year clinical factors were 0.622, 0.731 and 0.753, respectively (**Figures 8D–F**). The 1-, 3-, and 5-year AUC values for age, gender, AJCC stage, and tumor status are shown in **Figures 8A–C**.

### Validation of the Screened Genes by qRT-PCR and External Melanoma Database

**Figure 9:**
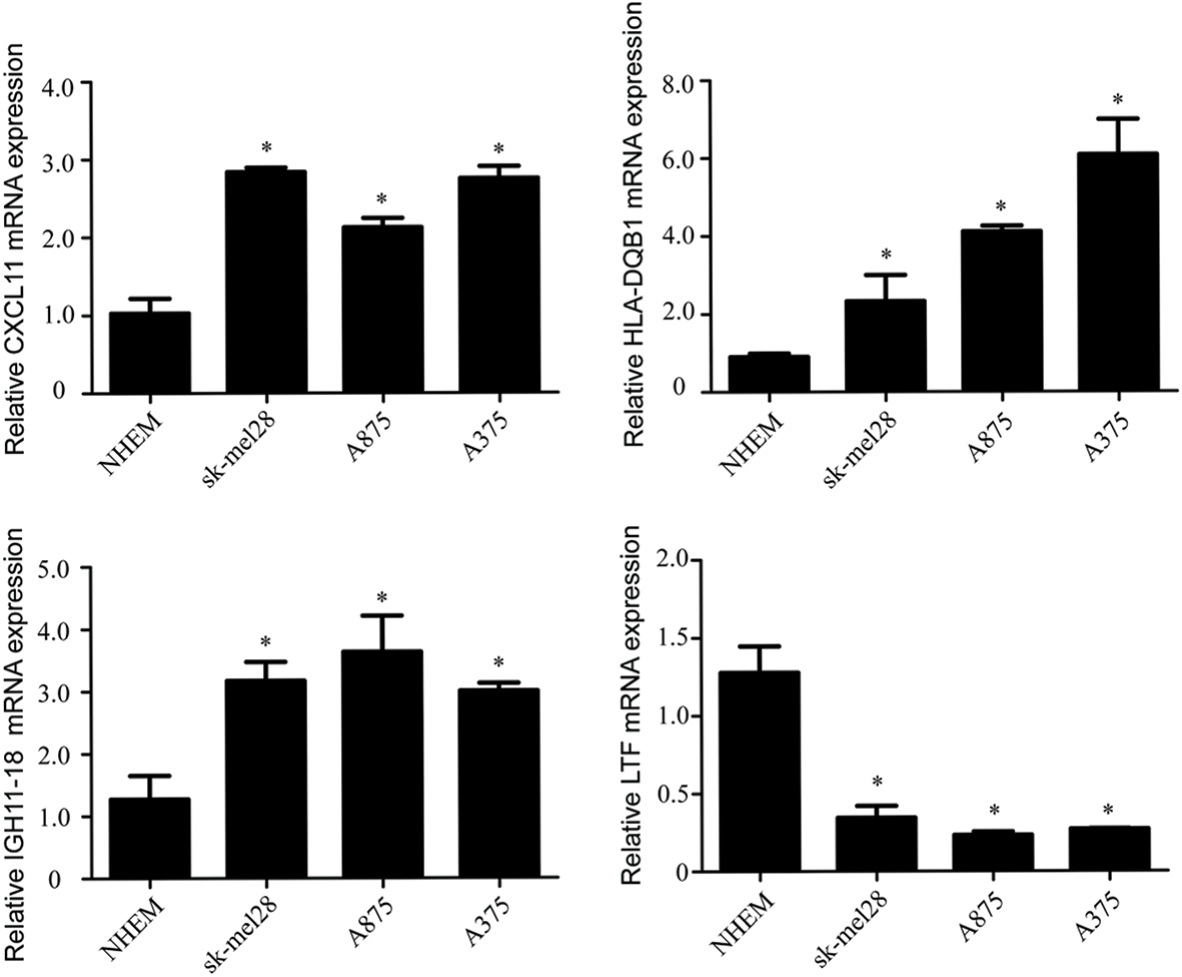
The mRNA levels of IGHV1-18, CXCL11, LTF and HLA-DQB1 in melanoma cell line A375, A815, SK-MEL-28 and NHEM. Data are expressed as mean ± SEM. *P < 0.05. NHEM, normal human epidermal melanocytes.

Compared with the normal melanocytes, IGHV1-18, CXCL11 and HLA-DQB1 were highly expressed in melanoma cell line A375, A815 and SK-MEL-28, and LTF was downregulated in melanoma cell line A375, A815 and SK-MEL-28 (**Figure 9**), and both had statistical significance (*P* < 0.05). And the stability of the identified prognostic immune-related genes were substantiated by the external validation dataset GSE15605 containing 58 melanoma samples. Consistent with previous results, the expression of CXCL11 was higher while LTF was lower in the melanoma samples compared with normal samples. (**Figure S1**).

### Correlation of the Identified Prognostic Immune-Related Genes With the Immune Cell Subtypes That Infiltrate Melanoma and the Molecular Subtypes of Cutaneous Melanoma

**Figure 10:**
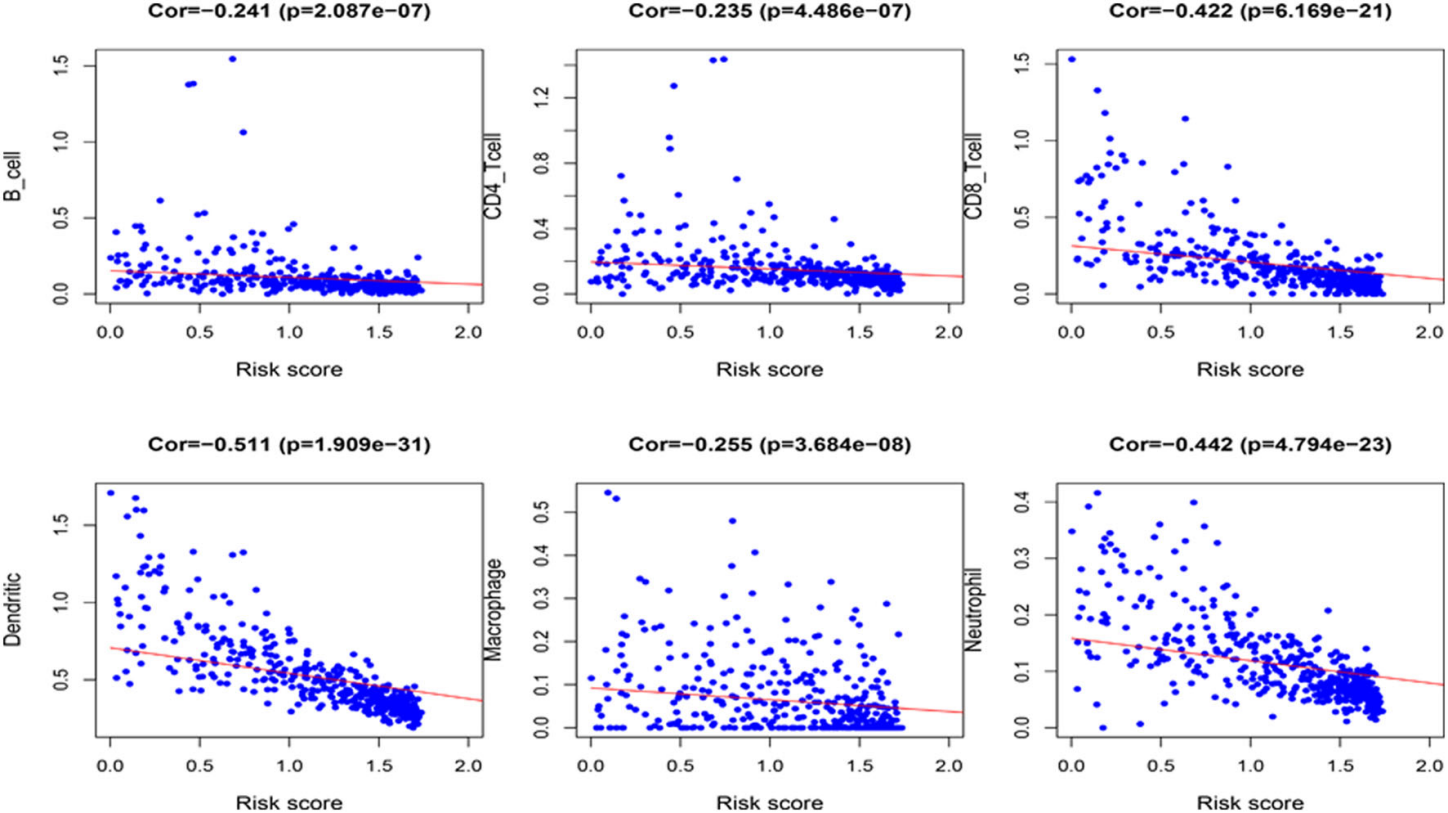
Correlation between the 4 immune-related genes prognostic signature for melanoma and the infiltration of immune cell subtypes. The six most relevant infiltration of immune cell subtypes are shown in the figure.

Because the 4 genes IGHV1-18, CXCL11, LTF and HLA-DQB1 are associated with tumor immunity, we used the TIMER database to analyze the correlation between the prognosis of these 4 genes and the infiltration of immune cell subtypes in melanoma (**Figure 10**). The correlation value of B cells with the risk score was −0.241, and the correlation value of CD4+ T cells with the risk score was −0.235. The correlation value of CD8+ T cells with the risk score was -0.422. The correlation values of dendritic cells with the risk score was −0.511. The correlation value of macrophages with the risk score was −0.255, and the correlation value of neutrophils with the risk score was −0.442. The above results suggest that the prognosis of melanoma is significantly negatively correlated with infiltration by these immune cell subtypes. In addition, compared with the normal control, the expression of IGHV1-18, CXCL11 and HLA-DQB1 were higher in the patients with cutaneous melanoma, while the expression of LTF was lower (**Figure S2**). We divide cutaneous melanoma into four subtypes (BRAF-mutant, NF1-deficient, NRAS-mutant and triple wild-type). We found that the expression of CXCL11 (*P* = 0.1), LTF (*P* = 0.28), and HLA-DQB1 (*P* = 0.67) had no significant relation to the subtypes of cutaneous melanoma through TISIDB database (**Figure S3**).

## Discussion

Melanoma is the most invasive form of skin cancer, and the incidence continues to rise worldwide. Although intense intermittent sun exposure is the main risk factor for melanoma, family history of melanoma, genetic susceptibility, environmental factors, and immunosuppression are other factors that affect the incidence (19). In recent years, immunotherapy and targeted therapy of specific factors have been increasingly used to treat melanoma. Liao et al. developed a predictive model based on two gene signatures including CCL8 and DEFB1 but lacked an exploration of its relationship with immune cells (20). Meng et al. established a signature consisted of 33 immune-related gene (IRG) pairs which associated with OS in malignant melanoma and analyzed the variations of the abundance of immune cells (21). Liu et al. identified 10 DE IRGs between primary and metastatic melanoma, and investigated the immune infiltration and tumor mutation burden in different risk groups (22).

In this study, we focused on the immune infiltrating status in melanoma and selected IGHV1-18, CXCL11, LTF and HLA-DQB1 from immune cell infiltration cluster as immune cell infiltration-related DEGs through the analysis of differences in melanoma samples and the construction of prognostic models. In addition, we further explored the correlation of the immune cell infiltration-related DEGs with the specific immune cell subtypes, which may provide more details for the exploration of the mechanisms by which DEGs regulate the development and prognosis of melanoma.

The CXCL9, -10, -11/CXCR3 axis is involved in inflammatory responses, leukocyte trafficking, adaptive resistance, hematopoiesis, cancer cell transfer and angiogenesis. Tokunaga et al. found that the CXCL9, CXCL10, and CXCL11/CXCR3 axis can be used as novel tumor treatment targets (23). C-X-C motif chemokine 11 (CXCL11) is regarded as the dominant CXCR3 agonist and can be induced by IFN-γ and type I interferons (24). CXCL11 has been found uniquely expressed in the melanoma with rich lymphocyte, and may play a potential role in the construction of tumor microenvironment by recruiting activated T-cells (25). Kremenovic et al. revealed that CXCL11, as a myeloid activation (MA) signature gene, had a positive correlation with the presence of M1 macrophages, mature dendritic cells (DC) and CD8^+^ T cells in cutaneous melanoma patients (26).

The lactoferrin (LTF) gene, located at 3p21.3, acts as a tumor suppressor gene in diverse tumors. Zhang et al. demonstrated that LTF is dysregulated in nasopharyngeal carcinoma cell lines (27). Yi HM and others discovered expression, genetic and epigenetic alterations of the LTF gene in nasopharyngeal carcinoma cell lines (28). Wei et al. found that in B16-F10 melanoma metastasis model, the metastatic rate was higher in the LTF knockout mice (29). LTF may play a protective role in melanoma metastasis by inducing differentiation and apoptosis of myeloid-derived suppressor cells (MDSCs) and up-regulating TLR9 expression.

Polymorphisms of human leukocyte antigen (HLA) genes are thought to be associated with the susceptibility to a variety of malignancies and involved in the progress of carcinogenesis, tumor proliferation and immune escape (30). HLA-DQB1 is more extensively studied in gastric cancer and cervical cancer (31, 32). HLA-DQB1 * 0301 has been reported to be closely associated with the risk of melanoma development and progression (33). As far as we know there are indeed few reports on IGHV1-18 in melanoma. IGHV1-18 is commonly expressed in normal B cells, and the tumor or inflammatory conditions can affect B cells, which may result in mutations in the heavy chain clone gene and influence the antibody gene family usage preference (34, 35). Although IGHV1-18 has not been reported in melanoma, current studies suggest that the dynamic balance of B cells and antibodies may be related to the occurrence, development and prognosis of melanoma. In melanoma, B-cells can be polarized to produce IgG4, which has low anti-tumor efficacy and may represent a possible mechanism of tumor escape (36). In addition, although it is generally believed that Ig is produced only by B lymphocytes, recent studies have reported that IgG can also be produced by non-B cells, such as epithelial cancer cells. For example, compared with normal epithelial cells, IgG from cancer cells often show unique V(D)J rearrangement or mutation hotspots (37). Therefore, further research on IGHV1-18 changes in melanoma patients may be helpful for the diagnosis and prognosis of melanoma. We have included this part of discussion in our revised manuscript accordingly.

Immunotherapy, along with surgery, radiation therapy and chemotherapy, is rapidly becoming the standard treatment for cancer. In recent years, it has been demonstrated in a variety of tumor types that the level of immune cell infiltration is inversely related to tumor purity but positively correlated with responsiveness to immune checkpoint inhibitors, which results in better prognosis and immune response (38, 39). Our results showed that the status of overall increased infiltrating immune cells in melanoma has the potential to predict clinical prognosis. Melanoma could be divided into ”hot” and “cold” status (enrich in or lack of immune cells infiltration), and the hot status is likely to correlate with antigen processing and higher expression of interferons, TNF and chemokines pathways (40). We further analyzed the infiltrating immune cell subtypes which correlated with the prognosis of melanoma. CD8+ cytotoxic T lymphocytes (CTLs) are the preferred tool for targeting tumors, and effective antitumor immunity also requires CD4+ T cells (41). Experiments have shown that CD8+ T cells and CD4+ T cells play a role in the treatment of breast cancer, colon cancer, etc. (42, 43), especially in melanoma (44, 45). Enhanced dendritic actin network formation is clearly proven to have an effect on melanoma (46). Samaniego R and others found that macrophage expression can predict human primary cutaneous melanoma progression (47). Protumor activities of macrophages have also been detected in the progression of melanoma (48). Forsthuber A and others found that CXCL5 played a role as a regulator of neutrophil function in cutaneous melanoma (49). Soler-Cardona A and others also confirmed that this mechanism is related to lymph node metastasis (50). The above results indicate that our screening and prediction about immune cell subtypes are reliable, which is beneficial to further research on melanoma immunotherapy.

Nevertheless, our study remains certain limitations. First, the data on which the prediction model was established were obtained from available public databases, though we validated it in melanoma cell lines through qRT-PCR and other external datasets, the immunohistochemistry staining of the protein level associated with DEGs and infiltrating immune cell in tumor tissues also deserves further validation. In addition, the immune cell types were identified by marker genes, but the expression level of them may not constant per cell, and hence, the cell number may be incompletely relevant to the expression level of marker genes (51). Further, a more comprehensive analysis of more types of immune cells and the stromal cells should be a focus of future research.

## Conclusion

In this study, by analyzing the differences between melanoma samples and immune cell infiltration data, we constructed a prognostic model and identified immune-related melanoma core genes. Relevant immune cell subtypes were also identified. In the future, the identified genes and subtypes may be used in targeted therapy and immunotherapy to provide new clinical treatment ideas.

## Data Availability Statement

The original contributions presented in the study are included in the article/**Supplementary Material**. Further inquiries can be directed to the corresponding authors.

## Author Contributions

J-AZ and X-YZ contributed equally to this work. J-AZ and MJ together with KC designed the experiment. DH, CL, and HG provided conceptual advice and critically reviewed the article. J-AZ, X-YZ and MJ together with KC conceptually designed the study and prepared the article. All authors contributed to the article and approved the submitted version.

## Funding

This study was supported by CAMS Innovation Fund for Medical Sciences (CIFMS-2017-I2M-1-017), the National Natural Science Foundation of China (grant nos. 81703152, 81872545 and 82073445), and the Jiangsu Province Natural Science Foundation (No. BK20170161).

## Conflict of Interest

The authors declare that the research was conducted in the absence of any commercial or financial relationships that could be construed as a potential conflict of interest.

## Supplementary Material

Supplementary Figure 1 The expression of CXCL11and LTF in melanoma patients from the GEO cohort (GSE15605), *P* < 0.001.

Supplementary Figure 2 The expression of CXCL11, LTF, and HLA-DQB1 in the group of cutaneous melanoma and normal control.

Supplementary Figure 3 Analysis of the expression of IGHV1-18, CXCL11, LTF, and HLA-DQB1 in different molecular subtypes of cutaneous melanoma.
