# Supplementary figures and images for: Development of an Immune-Related Gene Signature for Prognosis in Melanoma
Source: Front Oncol. 2021 Jan 21;10:602555. doi: 10.3389/fonc.2020.602555 (PMC7874014; doi:10.3389/fonc.2020.602555)

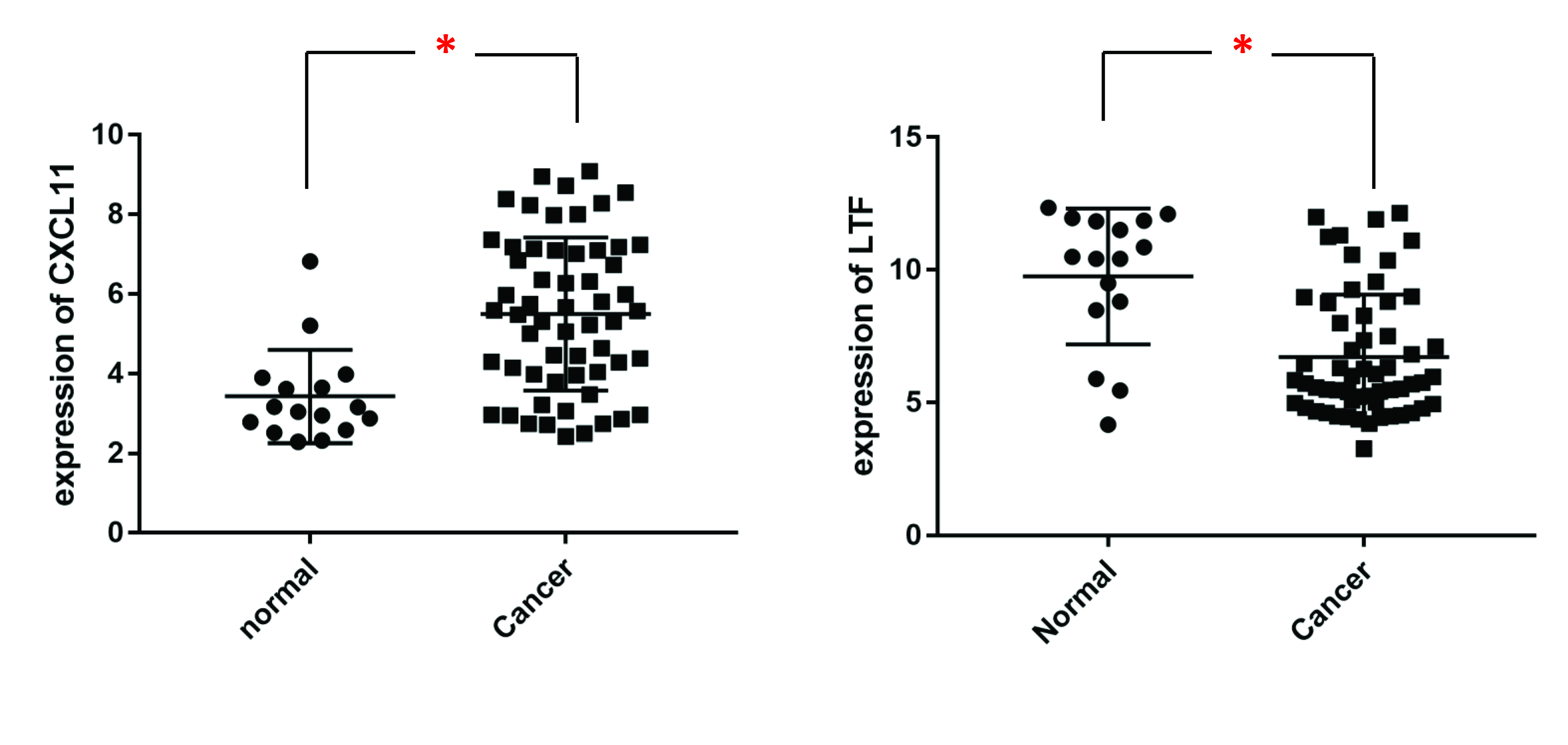

Supplement: Supplementary Figure 1 — The expression of CXCL11and LTF in melanoma patients from the GEO cohort (GSE15605), P < 0.001. [file Image_1.tiff]
